# Inhibition of microRNA-494-3p activates Wnt signaling and reduces proinflammatory macrophage polarization in atherosclerosis

**DOI:** 10.1016/j.omtn.2021.10.027

**Published:** 2021-11-04

**Authors:** Eva van Ingen, Amanda C. Foks, Tamar Woudenberg, M. Leontien van der Bent, Alwin de Jong, Philipp J. Hohensinner, Johann Wojta, Ilze Bot, Paul.H.A. Quax, Anne Yaël Nossent

**Affiliations:** 1 Department of Surgery, Leiden University Medical Center, 2300 RC Leiden, the Netherlands; 2 Einthoven Laboratory for Experimental Vascular Medicine, Leiden University Medical Center, 2300 RC Leiden, the Netherlands; 3 Division of BioTherapeutics, LACDR, Leiden University, 2333 CC Leiden, The Netherlands; 4 Department of Internal Medicine II, Medical, University of Vienna, 1090 Vienna, Austria; 5 Department of Laboratory Medicine, University of Vienna, 1090 Vienna, Austria; 6 Ludwig Boltzmann Institute for Cardiovascular Research, 1090 Vienna, Austria

**Keywords:** microRNA-494-3p, 14q32 locus, DLK1-DIO3 locus, antisense oligonucleotides, macrophage polarization, atherosclerosis, Wnt signaling, inflammation, cardiovascular disease, microRNA

## Abstract

We have previously shown that treatment with third-generation antisense oligonucleotides against miR-494-3p (3GA-494) reduces atherosclerotic plaque progression and stabilizes lesions, both in early and established plaques, with reduced macrophage content in established plaques. Within the plaque, different subtypes of macrophages are present. Here, we aimed to investigate whether miR-494-3p directly influences macrophage polarization and activation. Human macrophages were polarized into either proinflammatory M1 or anti-inflammatory M2 macrophages and simultaneously treated with 3GA-494 or a control antisense (3GA-ctrl). We show that 3GA-494 treatment inhibited miR-494-3p in M1 macrophages and dampened M1 polarization, while in M2 macrophages miR-494-3p expression was induced and M2 polarization enhanced. The proinflammatory marker CCR2 was reduced in 3GA-494-treated atherosclerosis-prone mice. Pathway enrichment analysis predicted an overlap between miR-494-3p target genes in macrophage polarization and Wnt signaling. We demonstrate that miR-494-3p regulates expression levels of multiple Wnt signaling components, such as LRP6 and TBL1X. Wnt signaling appears activated upon treatment with 3GA-494, both in cultured M1 macrophages and in plaques of hypercholesterolemic mice. Taken together, 3GA-494 treatment dampened M1 polarization, at least in part via activated Wnt signaling, while M2 polarization was enhanced, which is both favorable in reducing atherosclerotic plaque formation and increasing plaque stability.

## Graphical abstract

**Figure:**
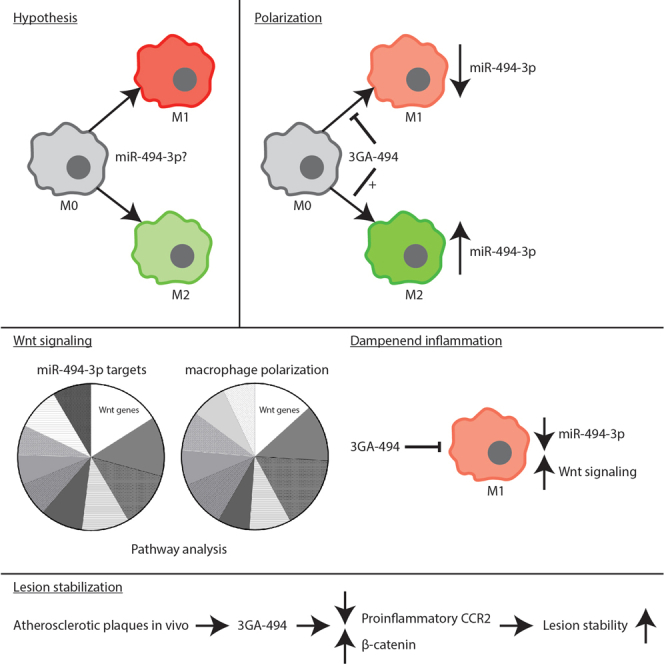

## Teaser

Treatment with Third-Generation Antisense against miR-494-3p (3GA-494) dampens proinflammatory M1 macrophage polarization, while anti-inflammatory M2 polarization is enhanced. Inhibition of miR-494-3p reduces the proinflammatory response in M1 macrophages, at least in part, via activated Wnt signaling. 3GA-494 could therefore be a potential therapeutic agent for stabilizing vulnerable atherosclerotic lesions.

## Introduction

Atherosclerosis is a chronic inflammatory disease characterized by formation of lipid-rich plaques in the arterial wall. Vulnerable plaques may eventually rupture and result in a cardiovascular event, such as myocardial infarction or ischemic stroke.^1^ Macrophages are cells of the innate immune system that play a central role in atherosclerosis. Circulating monocytes are recruited to the lesion site, where they differentiate into macrophages. Within the plaque, macrophages can polarize in response to signals from cytokines and chemokines, but also from bioactive lipids such as cholesterol and oxidized low-density lipoproteins (LDLs).^2^, ^3^, ^4^, ^5^, ^6^

*In vivo*, different subtypes of macrophages are present, each performing distinct functions. Historically, polarized macrophages were classified into M1 proinflammatory or M2 anti-inflammatory macrophages. *In vitro*, M1 macrophages polarize in response to interferon-γ (IFNγ) and lipopolysaccharide (LPS). M1 macrophages are considered to be potent effector cells that prime the immune system for action. Alternatively activated M2 macrophages polarize in response to interleukin-4 (IL-4) and IL-13. M2 macrophages induce an anti-inflammatory response whereby they counteract activation of the immune system.^7^, ^8^, ^9^, ^10^ The *in vitro* M1/M2 classification, however, is an oversimplification compared with the *in vivo* situation. Macrophage plasticity is highly dynamic, and macrophages continuously adapt to the signals they receive from their environment.^3^^,^^9^, ^10^, ^11^

As macrophages are exposed to diverse stimuli in the plaque, it is unlikely that pure M1 and M2 macrophages are present. However, markers for both M1 and M2 macrophages are present in plaques of mouse and human, with the M1 macrophage as the predominant phenotype.^12^, ^13^, ^14^, ^15^ The M1-like phenotype is associated with a proatherogenic response and located in rupture-prone, unstable regions. The M2-like phenotype is associated with an anti-atherogenic response and located in stable regions and the surrounding adventitial tissue.^4^^,^^16^^,^^17^ Because of their dynamic plasticity and key role in atherosclerosis, macrophages are an attractive therapeutic target to reduce inflammation and resolve atherosclerosis.

Several microRNAs have been described to regulate cellular pathways in macrophages, either by inhibiting or promoting inflammatory responses.^18^, ^19^, ^20^ MicroRNAs are short non-coding RNAs which regulate gene expression at the post-transcriptional level. MicroRNAs facilitate degradation of mRNA or inhibition of protein translation by binding to the 3′ untranslated region of their target mRNA.^21^ Because one microRNA has multiple target mRNAs, changes in microRNA expression can have a major impact on cellular processes, including complex signaling pathways.

A large non-coding RNA cluster located on the long arm of human chromosome 14, the 14q32 cluster (12F1 in mice), encodes more than 50 microRNAs. We have investigated inhibition of single 14q32 microRNAs in different models for vascular remodeling.^21^, ^22^, ^23^, ^24^, ^25^, ^26^ In murine models for intimal hyperplasia and early and advanced atherosclerosis, inhibition of 14q32 microRNAs, miR-494-3p in particular, resulted in smaller lesions with increased stability.^23^^,^^25^^,^^26^ In both intimal hyperplasia and advanced atherosclerosis, lesions contained fewer macrophages after miR-494-3p inhibition.^23^^,^^26^ Also, downregulation of miR-494-3p resulted in upregulation of miR-494-3p targets, such as IL-33, metalloproteinase inhibitor 3, and transforming growth factor β2, in the carotid artery.^25^ In addition, proatherogenic Ly6C^hi^ monocytes in the circulation were reduced when miR-494-3p was inhibited.^26^ Based on these results, we hypothesized that miR-494-3p directly influences macrophage polarization and activation.

Here, we aimed to investigate whether miR-494-3p directly influences macrophage polarization in atherosclerosis. We show that endogenous miR-494-3p expression is regulated during macrophage polarization *in vitro*. Also, miR-494-3p regulates mRNA and protein levels of key polarization markers in macrophages. Inhibition of miR-494-3p reduces the proinflammatory response in macrophages *in vitro* and reduces the proinflammatory marker CCR2 in atherosclerotic plaques *in vivo*. Pathway enrichment analysis predicted that miR-494-3p has more than 70 targets involved in macrophage polarization, with most of these involved in Wnt signaling. We confirmed that miR-494-3p targets components of the Wnt signaling pathway and that treatment with Third-Generation Antisense against miR-494-3p (3GA-494) activates Wnt signaling in cultured M1 macrophages. Also, in plaques of atherosclerotic mice, Wnt signaling appeared activated in response to 3GA-494 treatment.

## Results

### MiR-494-3p expression is regulated during macrophage polarization

**Figure 1:**
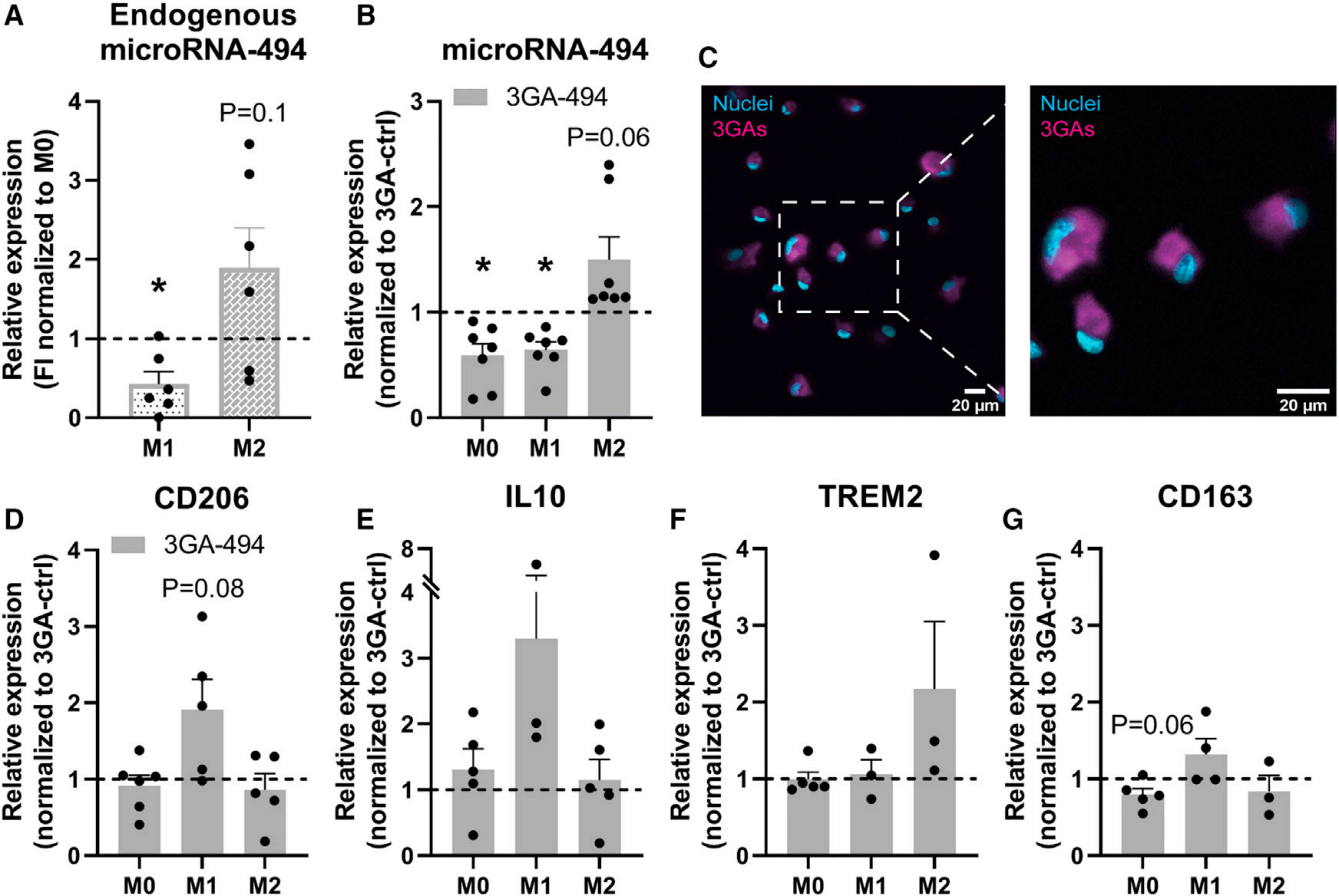
Expression of miR-494-3p and key polarization markers in human macrophages treated with 3GA-494 or 3GA-ctrl (A) Endogenous miR-494-3p expression in primary human macrophages during M1 and M2 polarization, shown as fold increase (FI) normalized to miR-494-3p expression in M0 macrophages for each donor. (B) MiR-494-3p expression in resting M0 and M1 and M2 polarized macrophages treated with 3GA-494, normalized to 3GA-ctrl treated M0, M1, and M2 macrophages, respectively. (C) M0 macrophages treated with IRDye-800-CW-labeled 3GA-494 for 24 h. Right image is a zoom-in image of the left image. Scale bars, 20 μm. Expression levels of (D) M2 markers cluster of receptors differentiation 206 (CD206), (E) interleukin-10 (IL10), (F) triggering receptor on myeloid cells 2 (TREM2), and (G) receptor for hemoglobin-haptoglobin complexes CD163 (N = 5). Expression levels in (D) to (G) were normalized to 3GA-ctrl. U6 was used as a reference gene. A one-sample t test was performed to compare single treatment with the control, within each individual donor. N is represented by the individual dots. Variations in N are caused by the exclusion criteria, as explained in materials and methods. Data are presented as mean ± SEM. ∗p < 0.05 compared with M0 (A) and 3GA-ctrl (B).

To study miR-494-3p inhibition in different macrophage subsets, we utilized *in vitro* polarized macrophages, isolated and differentiated from different individual human blood donors or from murine bone marrow, with either LPS/IFNγ for M1 or IL-4/IL-13 for M2 polarization. Macrophages polarized toward M1 showed decreased miR-494-3p expression compared with M0 macrophages (p = 0.02). In contrast, macrophages polarized toward M2 showed a trend toward increased miR-494-3p expression compared with M0 (p = 0.1; Figure 1A). Treatment with microRNA inhibitor 3GA-494 decreased miR-494-3p expression in M0 and M1 macrophages compared with 3GA-ctrl (p = 0.01 and p = 0.003, respectively), as expected. In contrast, in M2 macrophages, miR-494-3p expression appeared upregulated after 24 h of treatment with 3GA-494 (p = 0.06; Figure 1B). We have shown previously that expression of miR-494-3p upregulates in specific cell types and even whole tissues after treatment with 3GA-494,^26^ likely via an autoregulatory mechanism. 3GA-494-treated M2 macrophages also increased miR-494-3p secretion via extracellular vesicles (EVs) (p = 0.01), whereas in EVs from M0 and M1 macrophages no differences were observed in miR-494-3p secretion between 3GA-ctrl and 3GA-494, except for a general increased secretion by M1 macrophages treated with 3GA-494 for both miR-494-3p and U6 (p = 0.1 and p = 0.03, respectively; Figures S1A–S1C). Expression patterns of miR-494-3p in murine macrophages treated with 3GA-494 were not as clear as in human macrophages, but showed a similar trend in two out of three mice (Figure S1D). To confirm uptake of 3GAs by macrophages, we treated M0 macrophages with fluorescently labeled 3GA-494 (Figure 1C) and observed a strong fluorescent signal in the cytoplasm, as expected.

### MiR-494-3p regulates mRNA levels of key macrophage polarization markers

M1 and M2 polarization states are defined by expression of specific surface markers and secretory patterns.^2^, ^3^, ^4^ To confirm whether our polarization strategy by LPS/IFNγ or IL-4/IL-13 was successful, we measured expression levels of key polarization markers. In human macrophages, treatment with LPS/IFNγ resulted in upregulated expression of the M1 markers cluster of receptors differentiation 80 (CD80) (3GA-ctrl p = 0.04 and 3GA-494 p = 0.003), CD86 (3GA-ctrl p = 0.02 and 3GA-494 p = 0.02), and chemokine ligand 9 (3GA-ctrl p = 0.03 and 3GA-494 p = 0.04) (Figures S2A–S2C). In murine macrophages, expression of inducible oxide synthase (iNOS) appeared increased in M1 polarization (3GA-ctrl p = 0.1 and 3GA-494 p = 0.08) compared with M0 macrophages (Figure S1E). Treatment with IL-4/IL-13 resulted in a trend toward increased expression of the M2 marker mannose receptor CD206, an anti-inflammatory cytokine, in human macrophages compared with M0 (3GA-ctrl p = 0.1 and 3GA-494 p = 0.09; Figure S2E). IL-10 did not show differences compared with M0 in both groups and triggering receptor on myeloid cells 2 (TREM2) only showed increased expression in 3GA-494-treated human macrophages compared with M0 (3GA-494 p = 0.02; Figures S2F and S2G). In murine macrophages CD206 expression increased in response to IL-4/IL-13 treatment (3GA-ctrl p = 0.003, 3GA-494 p = 0.0004; Figure S1F).

Next, we investigated how altered miR-494-3p expression affects mRNA levels of key polarization markers in both M1 and M2 macrophage subsets in human macrophages. Expression levels of M1 markers were not different between 3GA-ctrl- and 3GA-494 M1-treated macrophages, except for the cytokine IL-1β, which showed a trend toward upregulation in 3GA-494 (p = 0.09; Figures S2A–S2E). However, expression of the M2 marker CD206 appeared increased in 3GA-494 M1 macrophages compared with 3GA-ctrl (p = 0.08; Figure 1D). Expression levels in M0 macrophages were not different between 3GA-494 and 3GA-ctrl, except for CD163, a receptor for hemoglobin-haptoglobin complexes, which showed a trend toward reduced expression in 3GA-494 M0 macrophages compared with 3GA-ctrl (p = 0.06; Figures 1G and S2H).

### 3GA-494 treatment reduces proinflammatory macrophage polarization *in vitro* and *in vivo*

Since macrophage M1 and M2 polarization states are defined by the presence of specific intracellular and surface proteins,^2^, ^3^, ^4^ we performed flow-cytometric analysis to further determine the effects of 3GA-494 treatment during M1 and M2 polarization. Expression of M1 markers C-C chemokine receptor 7 (CCR7) and CD86 and M2 marker CD206 was increased in M1 and M2 macrophages compared with M0, respectively, and confirmed polarization in human cells (p = 0.001 and p = 0.005, respectively; Figures S3A and S3B). In murine cells, intracellular expression of M1 marker iNOS and M2 marker Arginase-1 (Arg1) was increased in M1 and M2 macrophages compared with M0, respectively, and confirmed polarization in M1 and M2 subsets (p < 0.0001; Figures S3C and S3D).

**Figure 2:**
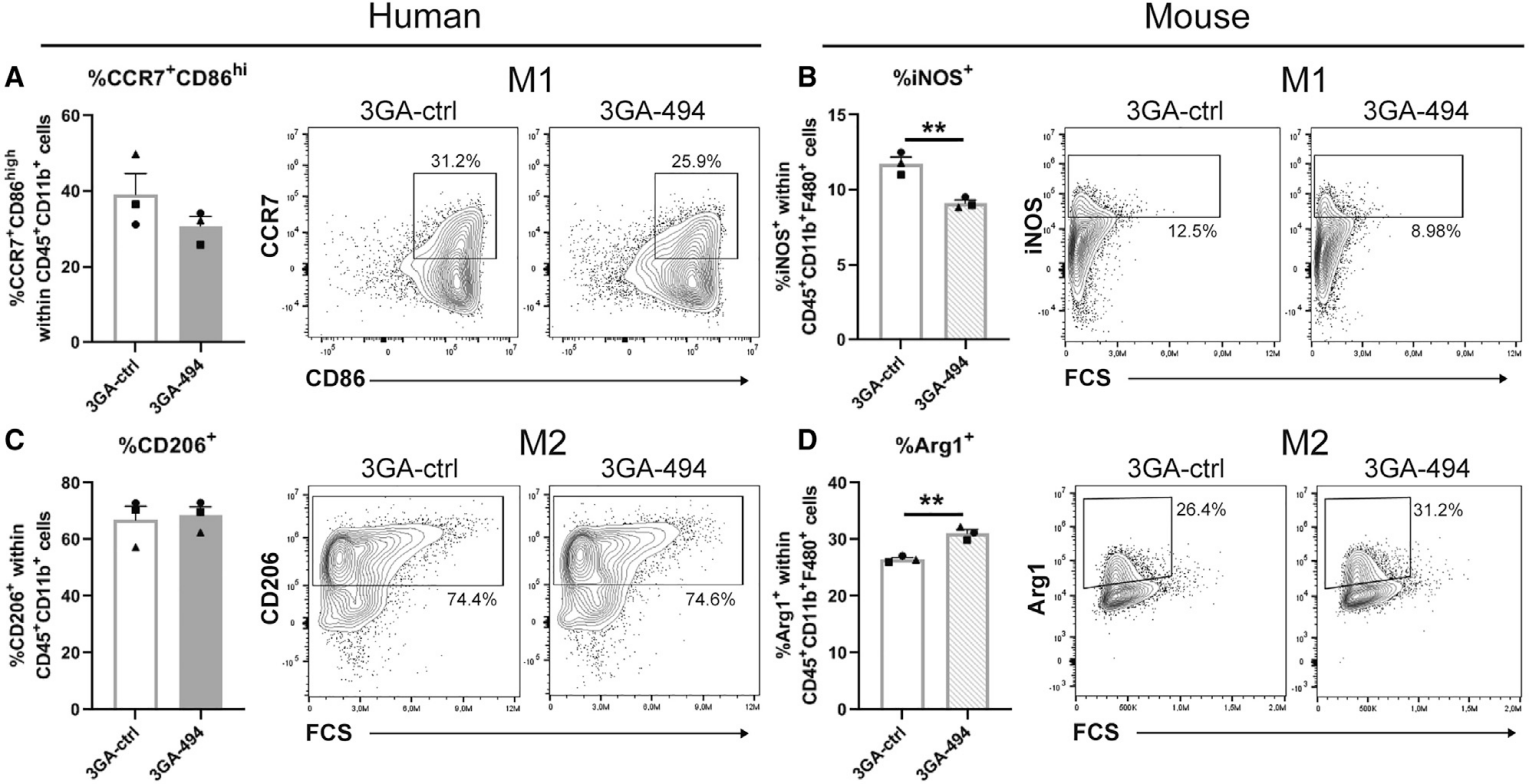
Flow-cytometric analysis of human and murine polarized macrophages treated with 3GA-494 or 3GA-ctrl Protein levels of M1 and M2 markers in human and murine *in vitro* polarized macrophages, treated with 3GA-494 or 3GA-ctrl for 24 h during polarization, analyzed by flow-cytometric analysis. (A) Percentage of M1 markers C-C chemokine receptor 7 (CCR7) and cluster of differentiation 86 (CD86)-positive cells in human M1 macrophages. (B) Percentage of M1 marker inducible oxide synthase (iNOS)-positive cells in murine M1 macrophages. (C) Percentage of M2 marker CD206-positive cells in human M2 macrophages. (D) Percentage of M2 marker Arginase-1 (Arg1)-positive cells in murine M2 macrophages. (A–D) Percentage (%) of positive cells, within live (A and C) CD45^+^CD11b^+^ or (B and D) CD11b^+^F4/80^+^ cells, in 3GA-ctrl- or 3GA-494-treated cells. Representative plots of both groups are shown. N is represented by the individual symbols. A two-tailed unpaired t test was performed to compare single treatment with the control. Data are presented as mean ± SEM. ∗∗p < 0.01 compared with 3GA-ctrl.

**Figure 3:**
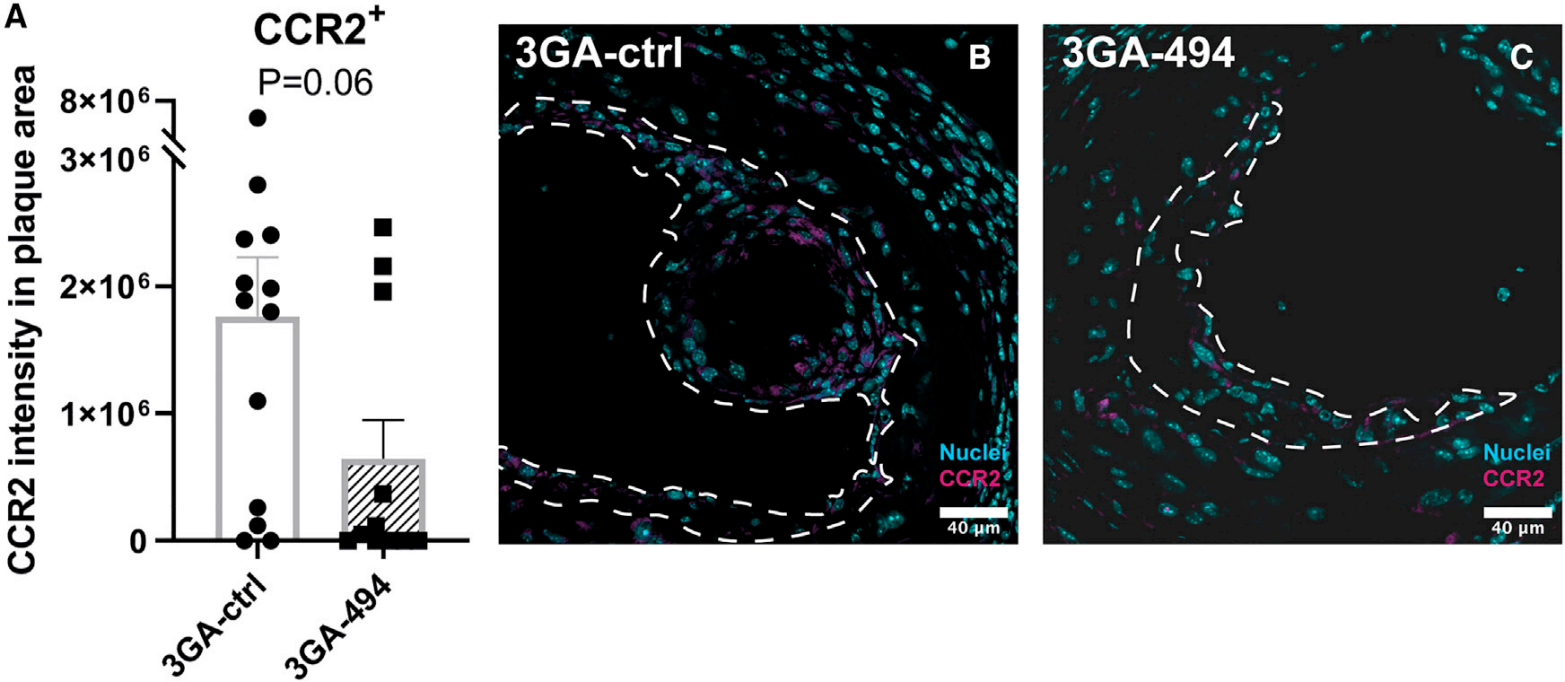
Proinflammatory marker CCR2 in carotid artery plaques of 3GA-494- or 3GA-ctrl-treated ApoE^–/–^ mice Immunofluorescence staining of proinflammatory marker C-C motif chemokine receptor-2 (CCR2). (A) Quantifications of CCR2 intensity in the plaque area (N = 13 and N = 11 in 3GA-ctrl and 3GA-494, respectively). Representative cross-sections of the carotid artery of mice treated with (B) 3GA-ctrl or (C) 3GA-494. Sections are stained with CCR2 (magenta) and Hoechst for nuclei (blue). Scale bars, 40 μm. Plaques are outlined with a dashed line. A two-tailed unpaired t test was performed to compare single treatment with the control. Data are presented as mean ± SEM.

Even though we did see differences in mRNA levels, we did not see differences between 3GA-494 and 3GA-ctrl in percentage of positive CCR7 and CD86 cells, CD206 cells, nor in the mean fluorescent intensity (MFI) per cell in human macrophages, possibly due to inter-donor variability (Figures 2A, 2C, and S3E–S3G). In murine M1 macrophages, however, the percentage of iNOS-positive cells was significantly decreased in 3GA-494 compared with 3GA-ctrl (p = 0.005; Figure 2B). In murine M2 macrophages, the percentage of Arg1-expressing cells was increased in 3GA-494 compared with 3GA-ctrl (p = 0.003; Figure 2D). The MFIs of iNOS and Arg1 were not different between groups (Figures S3H and S3I). 3GA-494 treatment thus attenuated M1 polarization in response to LPS/IFNγ stimulation and further increased M2 polarization in response to IL-4/IL-13, leading to an overall decrease in proinflammatory activity in murine macrophages. To confirm these findings *in vivo*, we stained for the proinflammatory M1 marker C-C motif chemokine receptor 2 (CCR2) in carotid artery plaques of hypercholesterolemic ApoE^–/–^ mice treated with 3GA-494 or 3GA-ctrl, as described previously.^25^ CCR2 intensity, quantified in the plaque area, appeared decreased in 3GA-494-treated mice compared with 3GA-ctrl mice (p = 0.06; Figure 3). This indicates that, in addition to the reduction in total plaque macrophages that we showed previously,^23^^,^^26^ the proinflammatory activity of intra-plaque macrophages may also be reduced *in vivo*.

### MiR-494-3p targets the Wnt signaling pathway

**Figure 4:**
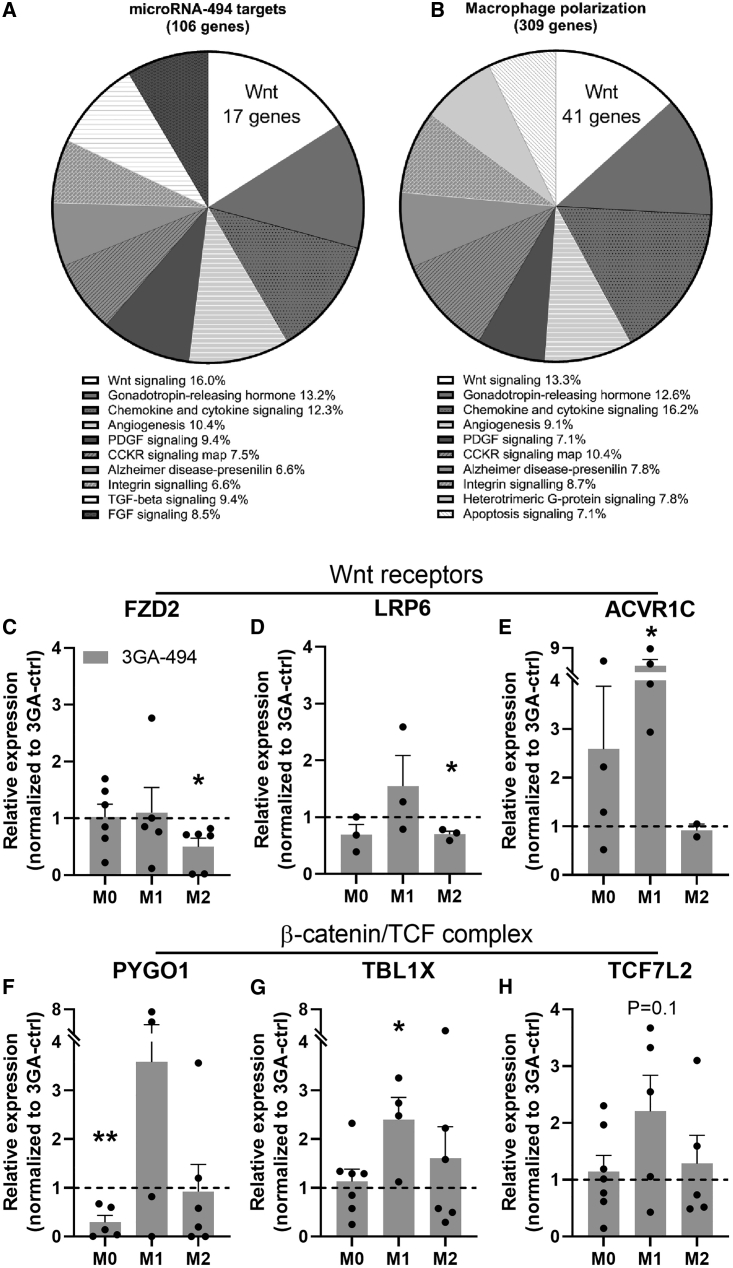
Pathway analysis and putative miR-494-3p target gene expression in the Wnt signaling pathway (A) A set of putative miR-494-3p targets and (B) genes from transcriptome analysis of M1 and M2 polarized macrophages were used in a pathway enrichment analysis. Top ten of pathways with most assigned genes are shown. Each part of the pie chart is represented as a percentage of the total genes in the top ten pathways. (C–H) Relative expression of genes, which are both putative miR-494-3p targets and components in the Wnt signaling pathway, in resting human M0, and polarized M1 and M2 macrophages, treated with 3GA-494 (N = 5) or 3GA-ctrl (N = 5). Wnt receptors (C) Frizzled class receptor 2 (FZD2), (D) LDL receptor-related protein 6 (LRP6), and (E) Activin A receptor type 1C (ACVR1C), and β-catenin/TCF complex genes (F) Pygopus homolog 1 (PYGO1), (G) transducing β-like 1 X-linked (TBL1X), and (H) transcription factor 7-like 2 (TCF7L2), in 3GA-494-treated macrophages compared with 3GA-ctrl. Expression levels were normalized to 3GA-ctrl. U6 was used as a reference gene. A one-sample t test was performed to compare single treatment with the control, within each individual donor. N is represented by the individual dots. Variations in N are caused by the exclusion criteria, as explained in materials and methods. Data are presented as mean ± SEM. ∗p < 0.05, ∗∗p < 0.01 compared with 3GA-ctrl.

To study the underlying mechanisms of miR-494-3p in macrophage polarization, we performed pathway enrichment analysis on a set of putative miR-494-3p targets, as predicted by Targetscan.org (v7.2) and a set of genes involved in M1 and M2 polarization, extracted from publicly available RNA-sequencing data.^27^ We found that 70 genes overlapped between both gene sets. The top ten of pathways containing most assigned genes is shown in Figures 4A and 4B. Out of ten pathways, eight overlapped between the two gene sets. Most putative miR-494-3p targets were assigned to the Wnt signaling pathway (16%; 17 genes in total, Figure 4A). Of the genes involved in macrophage polarization, 41 genes (13.3%) were also assigned to the Wnt signaling pathway (Figure 4B). Indeed, genes that were both putative miR-494-3p targets and components in the Wnt signaling pathway showed distinct expression patterns in each macrophage subset (Figures 4C–4H). Expression of Frizzled class receptor 2 and LDL receptor-related protein 6 (LRP6), both Wnt receptors, were significantly downregulated in 3GA-494-treated M2 macrophages compared with 3GA-ctrl (p = 0.02 and p = 0.007, respectively; Figures 4C and 4D). Activin A receptor type 1C was upregulated in 3GA-494-treated M1 macrophages (p = 0.03; Figure 4E). During canonical Wnt activation, a β-catenin/TCF complex is formed and translocated in the nucleus to induce transcription.^28^ Pygopus homolog 1 (PYGO1), transducing β-like 1 X-linked (TBL1X), and transcription factor 7-like 2 (TCF7L2) are all part of the β-catenin/T cell factor (TCF) complex.^29^, ^30^, ^31^ Inhibition of miR-494-3p significantly downregulated PYGO1 in M0 macrophages (p = 0.008; Figure 4F). TBL1X and TCF7L2 were upregulated, significantly or on trend (p = 0.05 [Figure 4G] and p = 0.1 [Figure 4H], respectively), in 3GA-494 M1 macrophages compared with 3GA-ctrl. Overall, 3GA-494 treatment resulted in increased target gene expression in M1 macrophages and decreased target gene expression in M2 macrophages, in accordance with the observed 3GA-494-induced miR-494-3p downregulation in M1 and miR-494-3p upregulation in M2 macrophages.

### MiR-494-3p inhibition activates Wnt signaling in M1 macrophages

**Figure 5:**
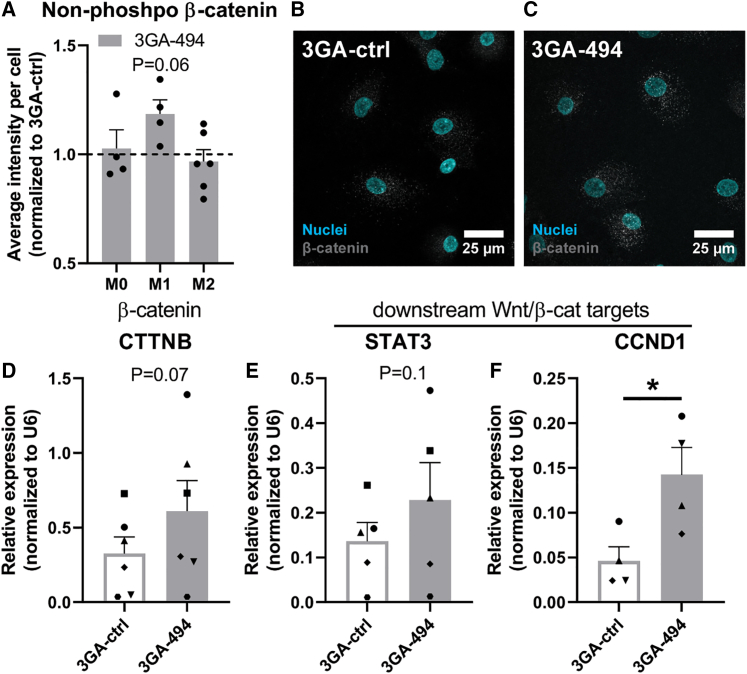
Active β-catenin and downstream Wnt target genes in human macrophages treated with 3GA-494 or 3GA-ctrl M0 and polarized M1 and M2 macrophages, treated with 3GA-494 or 3GA-ctrl, were stained with an antibody against the non-phosphorylated (non-phospho) form of β-catenin, the functionally active form in the canonical Wnt signaling. (A) Quantifications of the average non-phosho β-catenin intensity per cell. Representative images of M1 macrophages treated with (B) 3GA-ctrl or (C) 3GA-494. Non-phosho β-catenin is shown in gray and nuclei are stained with Hoechst, shown in blue. Relative expression of (D) β-catenin and two downstream Wnt transcription targets, (E) signal of transducer and activator of transcription 3 (STAT3), and (F) cyclin D1 (CCND1) in 3GA-ctrl or 3GA-494 M1 macrophages. U6 was used as a reference gene. A ratio paired t test was performed to compare single treatment with the control, within each individual donor. N is represented by the individual dots. Variations in N are caused by the exclusion criteria, as explained in materials and methods. Data are presented as mean ± SEM. ∗p < 0.05 compared with 3GA-ctrl.

We hypothesized that 3GA-494 treatment activates Wnt signaling in M1 macrophages and inhibits Wnt signaling in M0 and M2 macrophages. In canonical Wnt signaling, non-phosphorylated (non-phospho) β-catenin is translocated into the nucleus, where it forms a complex with TCF and induces transcription of downstream Wnt targets.^28^ Therefore, we also measured β-catenin and downstream Wnt targets, even though they were not direct targets of miR-494-3p, both by immunohistochemistry and by qRT-PCR. The amount of non-phosho β-catenin appeared increased (p = 0.06) in human M1 macrophages treated with 3GA-494 compared with 3GA-ctrl (Figures 5A–5C). In addition, gene expression levels of β-catenin and two downstream transcription targets, signal of transducer and activator of transcription 3 (STAT3) and cyclin D1, showed a trend toward or a significant upregulation in 3GA-494-treated M1 macrophages (p = 0.07, p = 0.1, and p = 0.02, respectively; Figures 5D–5F). This shows that the canonical Wnt signaling pathway was indeed activated upon miR-494-3p inhibition.

We did not observe effects on downstream Wnt activation by 3GA-494 in M0 or M2 macrophages, with the exception of STAT3, which was downregulated in M0 macrophages compared with 3GA-ctrl (p = 0.05; Figure S4), indicating that miR-494-3p acts in a cell-type-specific manner.

### 3GA-494 treatment activates Wnt signaling *in vivo*

**Figure 6:**
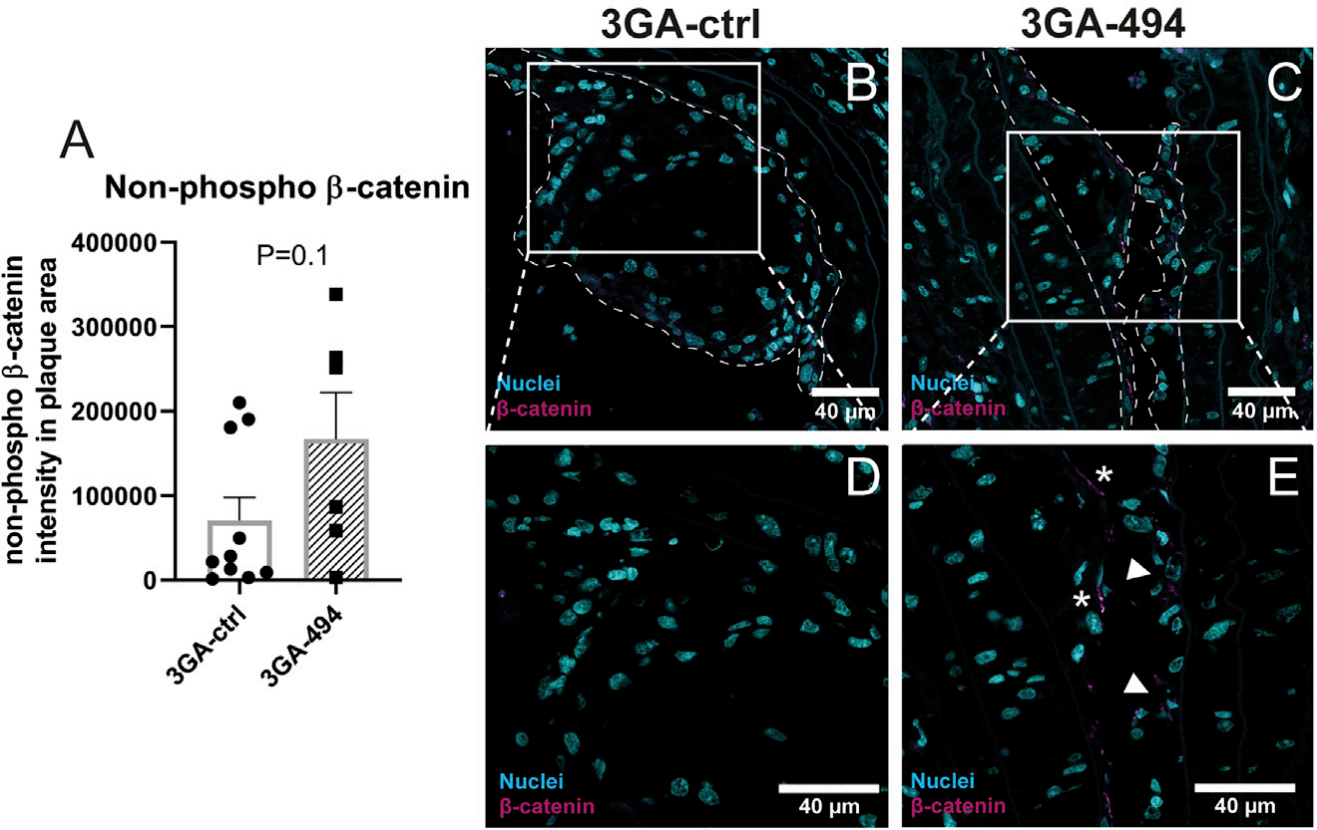
Active β-catenin in carotid artery plaques of 3GA-494- or 3GA-ctrl-treated ApoE^–/–^ mice Immunofluorescence staining of non-phosphorylated (non-phospho) β-catenin. (A) Quantification of non-phospho β-catenin intensity in the plaque area. Representative cross-sections of the carotid artery of mice treated with (B) 3GA-ctrl (N = 10) or (C) 3GA-494 (N = 6). Zoom-in images of (D) 3GA-ctrl- and (E) 3GA-494-treated mice. Asterisks point at non-phospho β-catenin-positive endothelial cells, which were excluded from the quantification analysis. Arrows point to non-phospho β-catenin-positive cells in the plaque area of a 3GA-494-treated mouse, which were included in the analysis. Non-phospho β-catenin is shown in magenta and Hoechst for nuclei in blue. Scale bars, 40 μm. Plaques are outlined with a dashed line. A two-tailed unpaired t test was performed to compare single treatment with the control. Data are presented as mean ± SEM.

To evaluate whether 3GA-494 treatment also leads to increased Wnt signaling in macrophages *in vivo*, we performed non-phospho β-catenin staining on plaques of ApoE^–/–^ mice treated with 3GA-ctrl or 3GA-494. We noticed that, particularly in 3GA-494-treated mice, non-phospho β-catenin was present in what are most likely endothelial cells lining the plaque (Figure 6C). Because our focus was on Wnt signaling in macrophages, we excluded the endothelial layer from the quantification. Some plaques from 3GA-494-treated mice were too small^25^ to perform quantification after exclusion of the endothelial layer, and these were excluded from the analysis completely. Mice treated with 3GA-494 showed a trend toward increased intra-plaque non-phospho β-catenin expression compared with 3GA-ctrl mice (p = 0.1; Figure 6), indicating that macrophage Wnt signaling also appears activated *in vivo* in response to 3GA-494 treatment.

In addition, we also stained for miR-494-3p with CD68 as macrophage marker and non-phospho β-catenin in human middle cerebral arteries from either a healthy, mildly atherosclerotic or a severely atherosclerotic section. Although this is purely anecdotal, non-phospho β-catenin and CD68 expression colocalized and expression of miR-494-3p increased in more advanced lesions (Figure S5).

## Discussion

In this study, we show that endogenous miR-494-3p expression is regulated during macrophage polarization and that miR-494-3p regulates mRNA and protein levels of key polarization markers in macrophages. Furthermore, inhibition of miR-494-3p reduced the proinflammatory response in cultured macrophages *in vitro*, and the proinflammatory marker CCR2 appeared reduced in atherosclerotic plaques *in vivo*. Finally, we show that miR-494-3p targets components of the Wnt signaling pathway and that 3GA-494 treatment leads to activated Wnt signaling in cultured M1 macrophages as well as apparent activation in plaques of atherosclerotic mice.

Our data show that miR-494-3p has a distinct role in each macrophage subtype, which becomes even more apparent upon treatment with 3GA-494. Both the increase in expression levels of anti-inflammatory receptors and cytokines and the reduction of M1 markers in M1 macrophages suggests that M1 polarization shifted toward a less inflammatory phenotype in response to miR-494-3p inhibition. This is consistent with the seemingly upregulated expression of STAT3, which suppresses immune responses in macrophages.^32^ M2 polarization was further promoted by 3GA-494 treatment, as the anti-inflammatory marker TREM2 and, in murine macrophages, expression of the M2 marker Arg1 were further increased. Dampening M1 polarization, while enhancing M2 polarization, is favorable in reducing both inflammation and atherogenesis. Indeed, the proinflammatory marker CCR2 appeared reduced in proatherogenic mice treated with 3GA-494 compared with 3GA-ctrl mice. We have demonstrated previously that plaque size decreased and plaque stability increased correspondingly upon 3GA-494 treatment.^25^^,^^26^ Likely, the subtle shift in macrophage polarization from proinflammatory toward anti-inflammatory contributed to this clinically advantageous phenotype.

We used primary human macrophages, differentiated from peripheral blood mononuclear cells (PBMCs), to more closely translate our results to a human clinical setting. Biological differences between donors, however, led to greater variations in the response to 3GA-494 treatment than in murine macrophages isolated and differentiated from mice with the same genetic background. Flow-cytometric analysis showed clear differences in polarization in response to 3GA-494 in murine macrophages, with decreased M1 iNOS and increased M2 Arg1 expression in M1 and M2 macrophages, respectively. Although human macrophages followed a similar pattern, the effects on polarization markers were more variable.

M2 polarization induced endogenous miR-494-3p expression, which was even further induced in response to 3GA-494 treatment. We have previously shown that miR-494-3p expression increased unexpectedly in response to treatment with the miR-494-3p inhibitor 3GA-494 in certain cell types and tissues, likely via autoregulatory mechanisms.^26^ In this study, we found that this phenomenon is even specific for differentially polarized subsets of the same cell type. MicroRNA processing can be regulated by RNA-binding proteins, which in turn are regulated by microRNAs themselves. Previously, we have demonstrated that the RNA-binding protein Mef2A directly binds to pri-miR-494-3p, for example.^24^ However, which precise mechanism underlies the miR-494-3p autoregulation in M2 macrophages remains to be determined.

Pathway enrichment analysis predicted that eight out of the top ten pathways overlapped between a set of 106 putative miR-494-3p target genes and a set of 309 genes directly involved in macrophage polarization. As we observed the greatest overlap in the Wnt signaling pathway, we focused on canonical Wnt signaling, but of course it is likely that other pathways, including chemokine and cytokine signaling, also play important roles in shaping macrophage phenotypes under the influence of miR-494-3p.

We demonstrate here that miR-494-3p targets mRNA levels of multiple components in the Wnt signaling pathway, all upstream of gene transcription induced by the β-catenin/TCF complex. MicroRNAs downregulate the expression of their target genes, consistent with our observations of miR-494-3p in M1 versus M2 macrophages. In M1 macrophages, where miR-494-3p expression is downregulated in response to 3GA-494, Wnt components were upregulated. In contrast, in M2 macrophages, where miR-494-3p is upregulated in response to 3GA-494, Wnt components were downregulated. It is noteworthy that different Wnt components appeared to be targeted by miR-494-3p in the two different macrophage subtypes. MicroRNAs have cell-type-specific target genes,^33^ which may help explain the distinct effects of 3GA-494 treatment on macrophage polarization within the two subtypes.

The Wnt signaling pathway has received little attention in the field of atherosclerosis so far, and is mostly known from cell development and differentiation and its role in diseases such as cancer.^28^ However, some studies suggest that Wnt signaling has a protective role against atherosclerosis.^34^, ^35^, ^36^, ^37^ The Wnt signaling pathway in macrophages has been described to be important for phagocytosis, clearance of LDLs, and foam cell formation, and thus may have a role in limiting cholesterol accumulation in atherosclerosis.^36^^,^^38^, ^39^, ^40^ WNT5A and LRP6 are both Wnt components that play a role in cholesterol metabolism,^39^^,^^40^ and both are putative targets of miR-494-3p. WNT5A expression levels did not respond to 3GA-494 treatment (Figure S2), but expression of LRP6 was significantly decreased in 3GA-494-treated M2 macrophages. Another putative miR-494-3p target, involved in cholesterol synthesis, is 3-hydroxy-3-methylglutaryl-coenzyme A synthase 1 (HMGCS1). HMGCS1 showed differential expression in three out of four human donors in both M1 and M2 macrophages (Figure S2). Finally, although not a direct target of miR-494-3p, TREM2, a marker for anti-inflammatory foamy lipid-laden macrophages involved in cholesterol metabolism, was increased in M2 macrophages in response to 3GA-494.^41^^,^^42^ These data suggest that miR-494-3p targets cholesterol metabolism, which corresponds to our findings in previous studies. For example, we have shown that in *in vitro* 3GA-494-treated macrophages, high-density lipoprotein-mediated efflux was increased compared with 3GA-ctrl-treated macrophages.^25^ Additionally, necrotic core sizes and plasma cholesterol levels in hypercholesterolemic mice treated with 3GA-494 were significantly reduced compared with 3GA-ctrl-treated mice.^25^^,^^26^ Precisely how the differential expression of Wnt targets in either macrophage subtype leads to altered cholesterol metabolism remains to be determined.

A surprising observation in this study was the apparent increase in non-phospho β-catenin staining in the endothelium lining the carotid artery plaques in mice treated with 3GA-494. Although we did not look into this in detail, endothelial β-catenin has been reported, via activation of Wnt signaling, to sustain endothelial integrity in atherosclerosis.^43^ Also, enforced expression of endothelial β-catenin reduces leakage of the blood-brain barrier.^44^ The apparently enhanced expression of endothelial β-catenin in 3GA-494-treated mice may indicate an improved endothelial barrier function, which would limit the development and progression of atherosclerosis even further.

It is a strength of this study that we were able to confirm the effects of 3GA-494 treatment in both murine and human primary macrophages and that we could link these effects to Wnt signaling for the first time, again in both mice and humans. To date, however, it remains technically challenging to direct 3GA-494 treatment to specific cell types or subsets. MiR-494-3p, for example, clearly has a distinct effect in the different macrophage subsets. This technical restriction has important implications for the potential future use of therapeutics that target miR-494-3p, but also microRNAs in general, as different effects in different tissues, cell types, and even cell subsets will have to be taken into account.

Taken together, 3GA-494 treatment inhibits miR-494-3p expression in M1 macrophages and dampens M1 polarization. Simultaneously, 3GA-494 treatment induces miR-494-3p expression and enhances M2 polarization, which is favorable in both reducing atherosclerotic plaque formation and increasing plaque stability. Furthermore, inhibition of miR-494-3p reduces a proinflammatory response in M1 macrophages, at least in part via activated Wnt signaling. 3GA-494 could therefore be a potential therapeutic agent for stabilizing vulnerable lesions and reducing the risk of a cardiovascular event such as myocardial infarction or ischemic stroke.

## Materials and methods

### Third-generation antisense

3GAs were designed with perfect reverse complementary to the mature target microRNA sequence and synthesized by Idera Pharmaceuticals (Cambridge, MA, USA). The same sequences of 3GAs (formerly named gene-silencing oligonucleotides) against miR-494 and a scrambled sequence control (3GA-ctrl) were used as described previously.^22^, ^23^, ^24^, ^25^, ^26^ For *in vitro* experiments, 3GAs were used at a concentration of 5 μg/mL medium. For *in vivo* experiments, a single dose of 1 mg/mouse in PBS was used.

### Isolation and differentiation of human macrophages

Blood was obtained from healthy volunteers according to recommendations of the medical ethical board of the Leiden University Medical Center and the Medical University of Vienna. All donors gave informed consent. PBMCs were isolated from whole blood using density gradient medium Lymphoprep (Stem Cell Technologies, Vancouver, Canada, #07581). CD14-positive cells (CD14^+^) were purified from the PBMCs using CD14^+^ Microbeads (MACS; Miltenyi Biotec, Bergisch Gladbach, Germany, #130-050-201). CD14^+^ cells were plated in 100-mm Petri dishes and cultured in RPMI containing L-glutamine (Gibco/Thermo Fisher Scientific, MA, USA, #11875093) supplemented with 25% heat-inactivated fetal calf serum (FCSi), 1% penicillin-streptomycin (P/S; Lonza, Basel, Switzerland, #DE17-602E) and 100 ng/mL mouse recombinant macrophage colony-stimulating factor (M-CSF; Peprotech, London, UK, #300-25). Medium was refreshed after 5 days. Ten days after isolation, differentiated resting macrophages (M0 macrophages) were washed with PBS and, subsequently, RPMI with all other reagents described above supplemented with 100 ng/mL IFNγ (Peprotech, #300-02) and 100 ng/mL LPS for proinflammatory M1, and 20 ng/mL IL-4 (Peprotech, #400-04) and 20 ng/mL IL-13 (Peprotech, #200-13) for anti-inflammatory M2 macrophages, were added for 24 h, as described previously.^7^ During M1 and M2 polarization, 3GA-494 or 3GA-ctrl at a concentration of 5 μg/mL medium was added to the media. M0 macrophages, without polarization cytokines added to the medium, were treated with 3GA-494 or 3GA-ctrl for 24 h after differentiation with M-CSF. Accutase was used to detach the cells (BD Biosciences, NJ, USA, #561527). Thereafter, cells were harvested and used for further analysis.

The macrophages from two donors did not respond to our polarization strategy and were excluded from all analyses. Due to low RNA yields in some (subsets) of the donors, mRNA levels appeared below the detection limit for some targets. Only donors that showed expression in both 3GA-ctrl and 3GA-494-treated macrophages were included in the analyses.

### Isolation and differentiation of mouse macrophages

Bone marrow cells were isolated from femurs and tibias of C57Bl/6 mice. After dissection, the bone marrow was flushed with PBS. Cells were filtered through a 70-μm cell strainer, centrifuged at 1,200 rpm for 10 min, and washed with PBS. The cell pellet was resuspended in ammonium-chloride-potassium lysis buffer (Gibco/Thermo Fisher Scientific #A1049201) and incubated on ice to lyse red blood cells. Subsequently, cells were centrifuged at 1,200 rpm for 10 min and washed twice with PBS. Cells were plated in a 100-mm Petri dish (Falcon, Corning, NY, USA, #353003) at a concentration of 8 × 10^6^ cells per dish. Cells were cultured in RPMI 1640 medium containing L-glutamine, supplemented with 25% FCSi and 1% P/S in a humidified incubator at 37°C. To obtain bone marrow-derived macrophages, cells were stimulated for 7–10 days with M-CSF (Peprotech, #315-02). Polarization conditions and 3GA treatments were the same as for human macrophages, but with murine cytokines IFNγ (Peprotech, #315-05), IL-4 (Peprotech, #214-04), and IL-13 (Peprotech, #210-13).

### Mice and experimental design

All animal experiments were performed in compliance with the Dutch government guidelines and the Directive 2010/63/EU of the European Parliament. As described previously,^25^ male ApoE^−/−^ mice, obtained from the local animal breeding facility (Gorlaeus Laboratories, Leiden University, Leiden, the Netherlands), were fed a Western type diet (WTD) for 6 weeks. Two weeks after the start of WTD, semi-constrictive collars were placed around both left and right carotid arteries to induce carotid artery plaque formation. At 4 and 18 days after surgery, mice received an intravenous injection via the tail vein of 1 mg/mouse and 0.5 mg/mouse (in 200 μL of PBS), respectively, of 3GA-494 or 3GA-ctrl. Four weeks after surgery, mice were sacrificed and carotid arteries were harvested for further analysis.

### RNA isolation and qRT-PCR

Total RNA was isolated by standard TRIzol (Thermo Fisher, #15596026) chloroform extraction. RNA concentration and purity were measured on the Nanodrop (Nanodrop Technologies).

For microRNAs, microRNA specific TaqMan qPRC kits (Thermo Fisher, #4427975) were used for reverse transcription and quantification by qPCR according to the manufacturer’s protocol. For mRNA, RNA was reverse transcribed using a “high-capacity RNA to cDNA” kit (Thermo Fisher, #4388950). SybrGreen reagents (Qiagen Benelux, Venlo, the Netherlands, #204145) were used for the qPCR. The data were normalized using a stably expressed endogenous control. U6 was used in human cells. In murine cells, miR-191 was used for microRNA normalization and Gapdh and ribosomal protein S18 for mRNAs. qPCR was performed on the VIIa7 PCR system (Applied Biosystems). A list of all primers used is shown in Table S1.

### Extracellular vesicles

Medium from 3GA-treated and simultaneously polarized macrophages was collected after 24 h of incubation and ultracentrifuged at 17,500 × *g* for 70 min to enrich for EVs (Beckman Coulter Optima XE-90 Ultracentrifuge). The lowest fraction containing most EVs was used for total RNA isolation using TRIzol liquid solution reagent (Thermo Fisher, #10296028) according to the manufacturer’s protocol.

### 3GA uptake

Human macrophages were seeded in chamber slides and treated with IRDye-800CW-labeled 3GA-494 (5 ng/μL; Idera Pharmaceuticals, Cambridge MA, USA) for 24 h. Thereafter, cells were washed twice with PBS and fixed with 1.5% formaldehyde. Nuclei were stained with DAPI and slides were embedded in ProLong Gold antifade (Invitrogen/Thermo Fisher, #P36930). Images were made under a Zeiss LSM700 confocal microscope.

### Flow-cytometric analysis

*In vitro* polarized macrophages, treated with 3GA-494 or 3GA-ctrl, were analyzed with flow cytometry to determine macrophage polarization phenotypes. Fc receptors were blocked using TruStain FcX (Biolegend) and an unconjugated anti-CD16/32 antibody (clone 2.4G2, BD Bioscience) for human and murine samples, respectively. Living cells were selected using Fixable Viability Dye eFluor 780 (1:2,000, eBioscience), and different cell populations were defined using anti-human and anti-mouse fluorochrome-conjugated antibodies. In human macrophages, the number of proinflammatory macrophages (CCR7^+^CD86^hi^) or anti-inflammatory macrophages (CD206^+^) was quantified and shown as a percentage of positive cells within live CD45^+^CD11b^+^ cells. For murine macrophages, intracellular iNOS and Arg1 were stained using transcription factor fixation/permeabilization concentrate and diluent solutions (BD Biosciences). The number of proinflammatory macrophages (iNOS^+^) or anti-inflammatory macrophages (Arg1^+^) was quantified and shown as a percentage of positive cells within live CD11b^+^F4/80^+^ cells. MFI per cell was also quantified. Fluorescence-activated cell sorting analysis was performed on a Cytoflex S (Beckman Coulter), and the acquired data were analyzed using FlowJo software.

### Collection of human middle cerebral arteries

Human middle cerebral arteries were collected from obduction material at the Department of Pathology of the Leiden University Medical Center. Collection, storage, and processing of the samples were performed in compliance with the Medical Treatment Contracts Act (WBGO, 1995) and the Code of Conduct for Healthy Research using Body Material (Good Practice Code, Dutch Federation of Biomedical Scientific Societies, 2002) and the Dutch Personal Data Protection Act (WBP, 2001). Arterial tissues were fixed in formaldehyde and embedded in paraffin.

### Immunofluorescence

For *in vitro* analyses, macrophages were seeded onto gelatin-coated glass coverslips on a 12-well plate. The next day, macrophages were polarized and/or treated with 3GAs. After 24 h, cells were washed with PBS, fixed with 4% paraformaldehyde, and again washed twice with PBS.

Murine tissues were fixed in formalin and embedded in paraffin. For murine *in vivo* analyses, paraffin sections (5 μm thick) of the carotid artery of 3GA-494- or 3GA-ctrl-treated mice were used. For both murine and human tissues, sections were dewaxed and antigen retrieval was performed prior to stainings.

For CCR2 staining, directly labeled goat anti-mouse CCR2 AF647 (Biolegend, CA, USA, #150604) was used to stain CCR2-positive cells. Nuclei were stained with Hoechst.

For non-phospho β-catenin staining in both mouse and human, primary non-phospho β-catenin (Ser33/37/Thr41) (D13A1) rabbit monoclonal antibody (Cell Signaling, MA, USA, #8814) with secondary donkey α-rabbit Alexa Fluor 647,was used. Anti-CD68 (Dako, #M0814—clone name: KP1) with secondary Alexa Fluor 555 DαMouse (Invitrogen, #A31570) was used to stain macrophages in human sections. Nuclei in murine sections were stained with oxazole yellow and in human sections with Hoechst.

After staining, slides were embedded in ProLong Gold antifade (Invitrogen, #P36930) and images were made under a Zeiss LSM700 confocal microscope. Fiji was used to perform immunofluorescence analysis.^45^ For murine *in vivo* sections, the plaque area was selected as the region of interest. Next, the integrated density, which is the sum of values of the pixels in the selected plaque area, was calculated. For *in vitro* analyses, the integrated density was calculated and normalized by the amount of nuclei.

### Fluorescence *in situ* hybridization of miR-494-3p

Fluorescence *in situ* hybridization (FISH) was used for detection of miR-494-3p expression and distribution. A protocol described by Chaudhuri et al., with some modifications, was used for FISH of miR-494-3p.^46^ In brief, formalin-fixed paraffin-embedded sections were dewaxed and an antigen retrieval step was performed. Next, sections were fixed in EDC (Sigma Aldrich, #E1769) in methylimidazole solution for 1 h. After washes with Tris-buffered saline (TBS), sections were prehybridized in 1× SSC buffer (Ultrapure SSC 20×; Thermo Fisher, #15557044) for 1 h at 37°C. One microliter of 10 μM miRCURY LNA microRNA detection probes against miR-494-3p or a scrambled sequence control (Qiagen, #339111) was added per 250 μL of hybridization buffer and heated at 65°C for 5 min to ensure denaturation. Probes were added to the sections and hybridized overnight at 37°C. After stringency washes at 42°C, sections were incubated in blocking buffer containing 1% bovine serum albumin (Sigma Aldrich, #B4287) and 3% normal goat serum in PBS for 1 h. Next, anti-digoxigenin-AP, Fab fragments (1:100; Roche, #11093274910) together with anti-α-smooth muscle actin (Dako, #M0851) were diluted in blocking buffer and incubated on the sections overnight at 4°C. The next day, after two washes in TBS, Hoechst and secondary antibody Alexa Fluor 555 DαMouse (Invitrogen, #A31570) in blocking buffer was added for 1 h at room temperature. After two washes in TBS, Cy5 from the Cy5-TSA kit (Perkin Elmer, #NEL745E001KT) was diluted in the provided buffer (1:100) and incubated on the sections for 10 min. Finally, sections were embedded in ProLong Gold antifade (Invitrogen, #P36930). Pictures were made under a Zeiss LSM700 confocal microscope.

### Pathway analysis

Two datasets of genes were used in the pathway analysis using PANTHER 16.0. A list of putative miR-494-3p targets, 623 genes in total, was generated using Targetscan.org (v7.2). A list of top differentially expressed genes from RNA-sequencing data comparing proinflammatory and anti-inflammatory macrophages, 2,200 genes in total, performed by Gerrick et al.,^27^ was used to select genes involved in macrophage polarization. Of these genes, 275 and 926 of miR-494-3p putative targets and genes in macrophage polarization, respectively, were assigned to a pathway. Next, the top ten pathways containing most assigned genes were selected, with in total 106 and 309 genes of miR-494-3p putative targets and macrophage polarization genes, respectively.

### Statistical analysis

Results are expressed as mean ± SEM. A two-tailed Student's t test was used to compare a single treatment group with the control group. For data normalized to 3GA-ctrl, a one-sample t test was performed. p < 0.05 was considered significant and p < 0.1 was considered a trend. A Grubbs' test was used to identify significant outliers (α < 0.05).

## Supplemental information

Document S1. Figures S1–S5 and Table S1

Document S2. Article plus supplemental information
