# Supplementary material for: Inhibition of microRNA-494-3p activates Wnt signaling and reduces proinflammatory macrophage polarization in atherosclerosis
Source: Mol Ther Nucleic Acids. 2021 Nov 4;26:1228–39. doi: 10.1016/j.omtn.2021.10.027 (PMC8607137; doi:10.1016/j.omtn.2021.10.027)
Supplement: Document S1. Figures S1–S5 and Table S1 [file mmc1.pdf]

OMTN, Volume *26*

Supplemental information

Inhibition of microRNA-494-3p activates Wnt signaling and reduces proinflammatory macrophage polarization in atherosclerosis

Eva van Ingen, Amanda C. Foks, Tamar Woudenberg, M. Leontien van der Bent, Alwin de Jong, Philipp J. Hohensinner, Johann Wojta, Ilze Bot, Paul.H.A. Quax, and Anne Yaël Nossent

Supplemental Data

Table 1. Primer sequences

**Wnt signaling**	
CTNNB1-HSA-FW	CTTGGCTATTACGACAGACTGC
CTNNB1-HSA-RV	ATTTACTAAAGCTTGGGGTCCA
CCND1-HSA-FW	GGAGACCATCCCCCTGAC
CCND1-HSA-RV	CCACTTGAGCTTGTTCACCA
STAT3-HSA-FW	GTGACAGCTTCCCAATGGAG
STAT3-HSA-RV	AACACCAAAGTGGCATGTGA
FZD2-HSA-FW	CCCGACTTCACGGTCTACAT
FZD2-HSA-RV	TCCACGAGTGCAGCGTCT
PYGO1-HSA-FW	AGGTGTACAACTAGGAAGCCC
PYGO1-HSA-RV	TTTGGATTCGGTGGTGGAGC
TBL1X -HSA-FW	TGGATCCTTCGACAAGTGCG
TBL1X -HSA-RV	GTTCCAGCACACCTCGAAGA
TCF7L2 -HSA-FW	AGGAGGATTCAGACACCCCT
TCF7L2 -HSA-RV	CCGTCGTGTGTAGCGTATGA
ACVR1C-HSA-FW	CTGTTGGTCTGGTTTACTGGGA
ACVR1C-HSA-RV	ATCGAGGGATCTGAAGGCAC
WNT5A-HSA-FW	GCCAGTATCAATTCCGACATCG
WNT5A-HSA-RV	TCACCGCGTATGTGAAGGC
**Cholesterol**	
HMGCS1-HSA-FW	GGCGTCCCACTCCAAATGAT
HMGCS1-HSA-RV	GTGGCAGGGAGTCTTGGTAC
**Polarization**	
CD80-HSA-FW	ACCTGGCTGAAGTGACGTTA
CD80-HSA-RV	TCCAGAGGTTGAGCAAATTATCC
CD86-HSA-FW	TTCCCTGATGTTACGAGCAAT
CD86-HSA-RV	CCAAGGAATGTGGTCTGGGG
CD206-HSA-FW	TCGGGTTTATGGAGCAGGTG
CD206-HSA-RV	TGAACGGGAATGCACAGGTT
IL1B-HSA-FW	AGCTACGAATCTCCGACCAC
IL1B-HSA-RV	CGTTATCCCATGTGTCGAAGAA
CXCL9-HSA-FW	CCAGTAGTGAGAAAGGGTCGC
CXCL9-HSA-RV	AGGGCTTGGGGCAAATTGTT
CD163-HSA-FW	TTTGTCAACTTGAGTCCCTTCAC
CD163-HSA-RV	TCCCGCTACACTTGTTTTCAC
IL-10-HSA-FW	TCAAGGCGCATGTGAACTCC
IL-10-HSA-RV	GATGTCAAACTCACTCATGGCT

TREM2-HSA-FW	GGTGGCACTCTCACCATTACG
TREM2-HSA-RV	CTCGAAGCTCTCAGACTCCC
CD206-MMU-FW	GTTCGGGATTGTGGAGCAGA
CD206-MMU-RV	ACAGACGGCTCCATTTGCAT
iNOS-MMU-FW	AGGGACAAGCCTACCCCTC
iNOS-MMU-RV	CTCATCTCCCGTCAGTTGGT
Arg-1-MMU-FW	CTCCAAGCCAAAGTCCTTAGAG
Arg-1-MMU-RV	AGGAGCTGTCATTAGGGACATC
**Housekeeping**	
U6-MMU/HSA-FW	AGAAGATTAGCATGGCCCCT
U6-MMU/HSA-RV	ATTTGCGTGTCATCCTTGCG
GAPDH-MMU/HSA-FW	AGAAGATTAGCATGGCCCCT
GAPDH-MMU/HAS-RV	ATTTGCGTGTCATCCTTGCG
RSP18-MMU-FW	TGCGAGTACTCAACACCAACA
RSP18-MMU-RV	GGTGAGGTCGAGTCTGCTTT

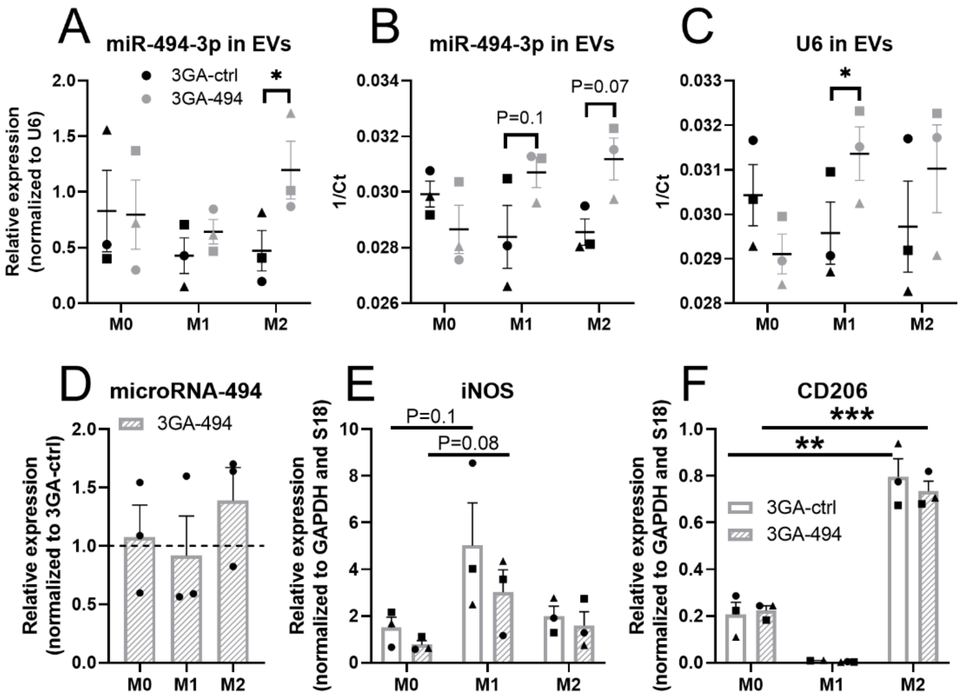

Supplemental Figure 1. Secretion of microRNA-494-3p in human extracellular vesicles and microRNA-494-3p expression and polarization markers in murine macrophages. (A-C) Expression of miR-494-3p in extracellular vesicles (EVs) secreted from M0, M1 and M2 human macrophages, treated with 3GA-ctrl or 3GA-494. (A) Relative expression of miR-494-3p normalized to U6. (B) 1 divided by absolute Ct value (1/Ct) of miR-494-3p and (C) 1/Ct of U6. (D) MiR-494-3p expression in resting M0 and polarized M1 and M2 murine macrophages treated with 3GA-494, normalized to 3GA-ctrl treated M0, M1 and M2 macrophages, respectively (N=3). Expression levels were normalized to 3GA-ctrl. MiR-191 was used as a reference gene. (E) Expression levels of M1 marker inducible oxide synthase (iNOS) and (F) M2 marker cluster of differentiation 206 (CD206) in M0, M1 and M2 macrophages (N=3). A two-tailed unpaired t-test was performed to compare single treatment with the control (3GA-ctrl or M0). (D-F) GAPDH and ribosomal protein S18 were used as a reference gene. Data are represented as mean ±SEM. ***P<0.001, **P<0.01, *P<0.05, compared to 3GA-ctrl or M0.

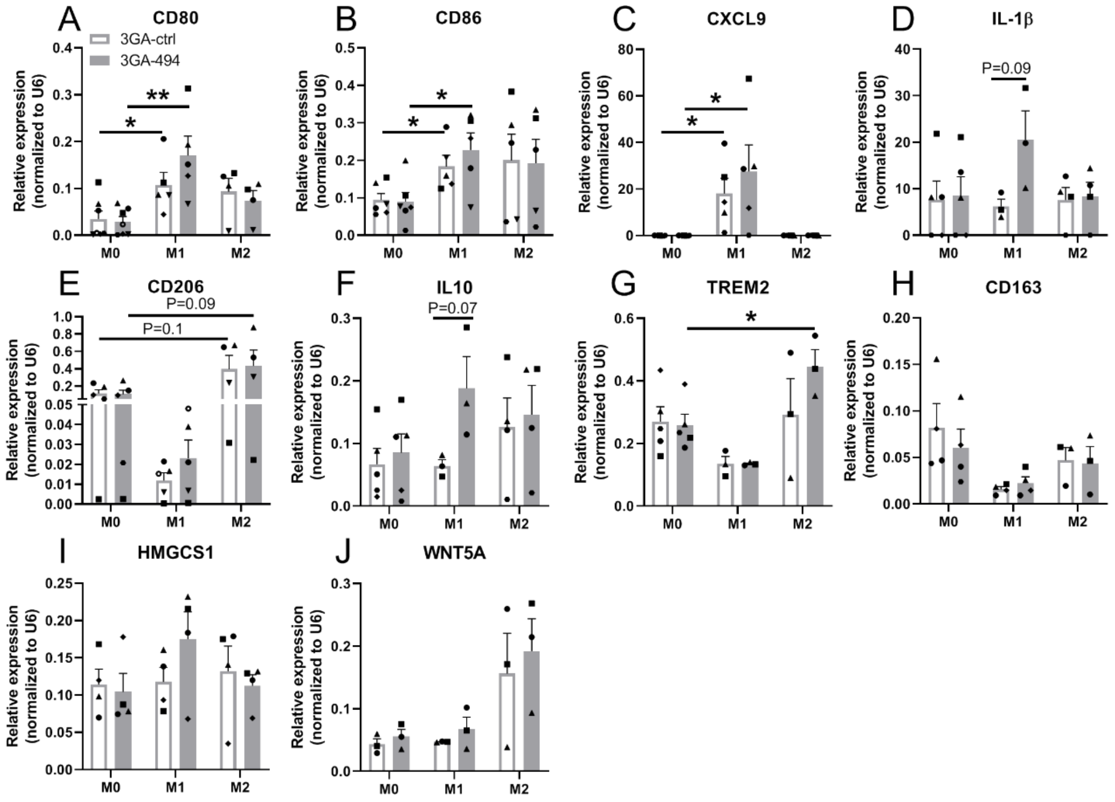

Supplemental Figure 2. Expression of key M1 and M2 polarization markers in human macrophages treated with 3GA-494 or 3GA-ctrl. Resting M0 and polarized M1 and M2 macrophages treated with 3GA-494 or 3GA-ctrl for 24 hours. Expression levels of M1 markers (A) cluster of differentiation (CD)80, (B) CD86, (C), chemokine ligand 9 (CXCL9) and (D) interleukin 1-β and expression levels of M2 markers (E) CD206, (F) interleukin 10 (IL10) and (G) triggering receptor on myeloid cells 2 (TREM-2). (H) Expression of CD163, a receptor for hemoglobin-haptoglobin complexes, (I) 3-Hydroxy-3-Methylglutaryl-CoA Synthase 1 (HMGCS1) and (J) Wnt family member 5A (WNT5A). A two-tailed unpaired t-test was performed to compare single treatment with the control (3GA-ctrl or M0). N is represented by the individual symbols. Variations in N are caused by the exclusion criteria, as explained in the material and methods. U6 was used as a reference gene. Data are represented as mean ±SEM. **P<0.01, *P<0.05, compared to 3GA-ctrl or M0.

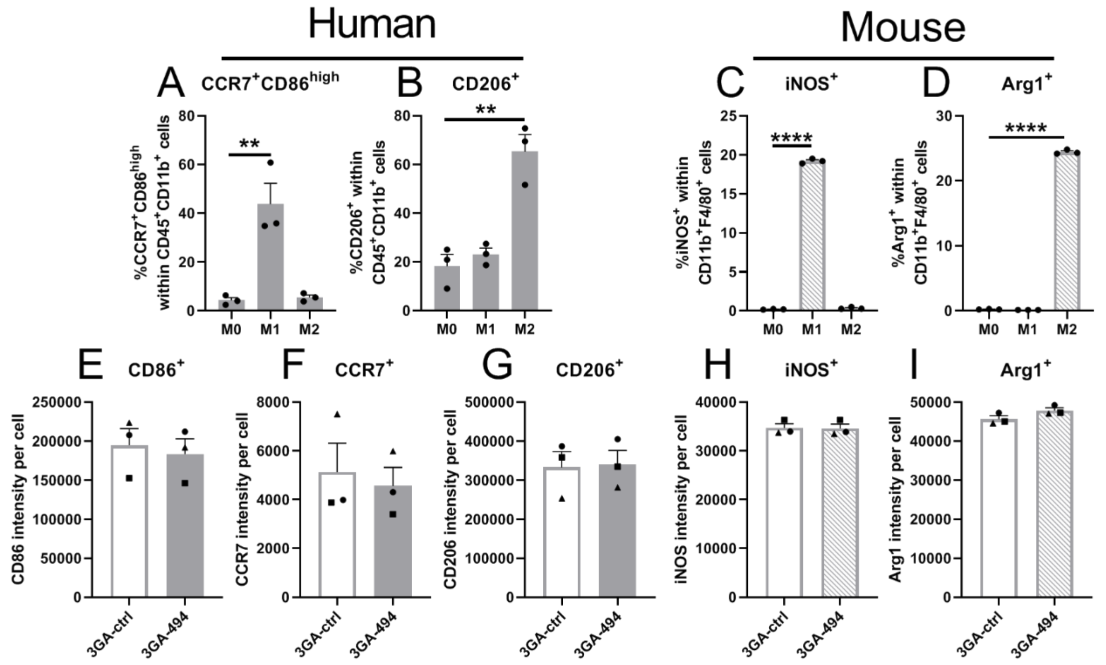

Supplemental Figure 3. Flow cytometric analysis of M1 and M2 markers in human and murine polarized macrophages. Protein levels of M1 and M2 markers in human and murine *in vitro* polarized macrophages (N=3). M1 polarization was induced with LPS and IFNγ. M2 polarization was induced with IL4 and IL13. Percentage of M1 markers (A) C-C chemokine receptor 7 (CCR7) and cluster of differentiation 86 (CD86) positive cells and percentage of M2 marker (B) CD206 positive cells in human M0 and polarized M1 and M2 macrophages. Percentage of M1 marker (C) inducible oxide synthase (iNOS) positive cells and (D) percentage of M2 marker Arginase-1 (Arg1) positive cells in murine M0 and polarized M1 and M2 macrophages. (E) CD86 and (F) CCR7 mean fluorescence intensity (MFI) per cell in human M1 macrophages and (G) CD206 MFI per cell in human M2 macrophages. (H) iNOS MFI in murine M1 macrophages and (I) Arg1 MFI in murine M2 macrophages. (A-D) Percentage (%) of positive cells within alive (A and B) $CD45^{+}CD11b^{+}$ or (C and D) $CD11b^{+}F4/80^{+}$ cells is shown. (E-I) MFI per cell, treated with 3GA-ctrl or 3GA-494. A two-tailed unpaired t-test was performed to compare single treatment with the control (M0). Data are represented as mean ±SEM. ****P<0.0001, **P<0.01, compared to M0.

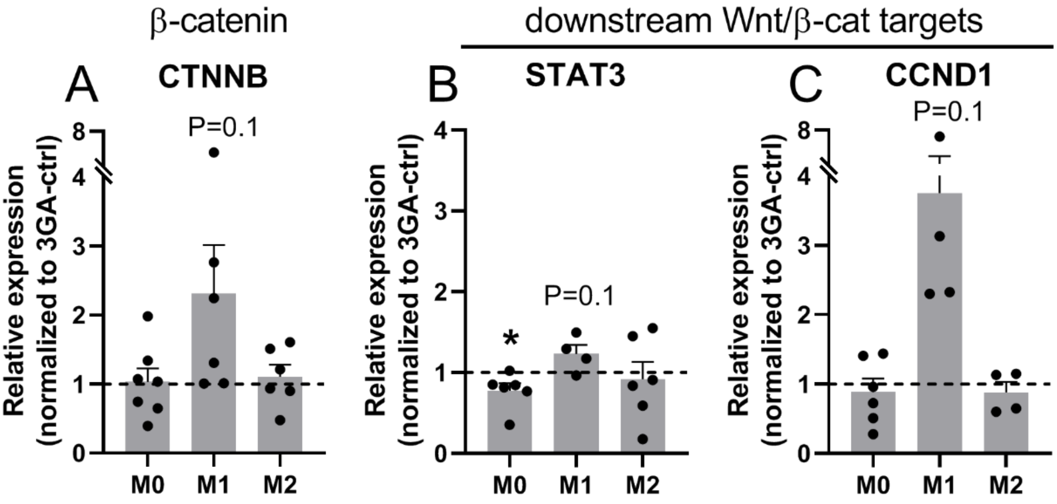

Supplemental Figure 4. Active β-catenin and downstream Wnt target genes in human macrophages treated with 3GA-494 or 3GA-ctrl. Relative expression levels of (A) β-catenin and two downstream Wnt transcription targets, (B) signal of transducer and activator of transcription 3 (STAT3) and (C) cyclin D1 (CCND1) in 3GA-ctrl or 3GA-494 treated M0 and polarized M1 and M2 human macrophages. Expression levels are normalized to 3GA-ctrl (1). U6 was used as a reference gene. A one-sample t-test was performed to compare single treatment with the control, within each individual donor. N is represented by the individual dots. Variations in N are caused by the exclusion criteria, as explained in the material and methods. Data are represented as mean ±SEM. *P<0.05, compared to 3GA-ctrl.

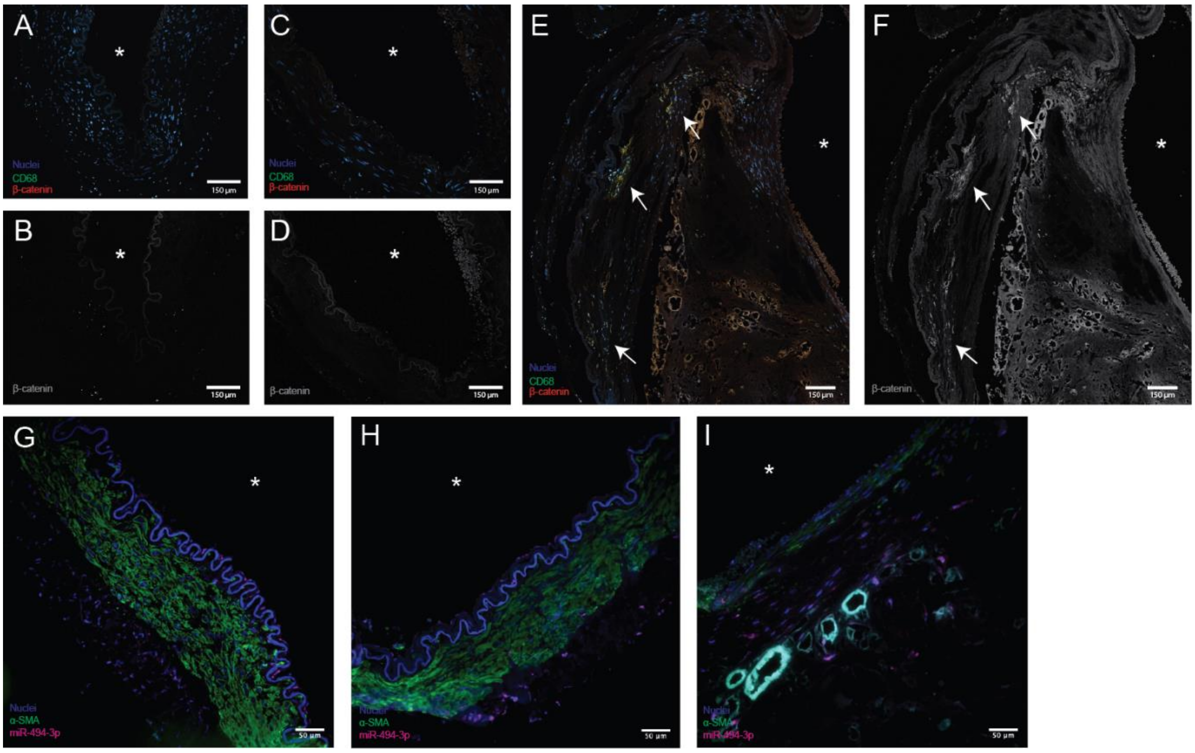

Supplemental Figure 5. Active β-catenin, CD68 and miR-494-3p in human middle cerebral arteries from either a healthy, mildly atherosclerotic or severely atherosclerotic section. (A, B) Healthy, (C,D) mildly atherosclerotic and (E,F) advanced atherosclerotic sections. (A, C, E) Sections were stained with an antibody against CD68 to stain for macrophages (green), the non-phosphorylated (non-phospho) form of β-catenin (red) and nuclei (blue). (B, D, F) β-catenin (grey) staining alone. (G) Healthy, (H) mildly atherosclerotic and (I) advanced atherosclerotic sections were stained with an antibody against α-smooth muscle actin (α-SMA; green) and with fluorescent in situ hybridization to stain for miR-494-3p (red). Nuclei are shown in blue. Arrows point at areas with both β-catenin and CD68 expression. Asterisks indicate the vessel lumen.
